# *Chlamydia trachomatis* Infection Induces Replication of Latent HHV-6

**DOI:** 10.1371/journal.pone.0061400

**Published:** 2013-04-19

**Authors:** Bhupesh K. Prusty, Christine Siegl, Petra Hauck, Johannes Hain, Suvi J. Korhonen, Eija Hiltunen-Back, Mirja Puolakkainen, Thomas Rudel

**Affiliations:** 1 Biocenter, Chair of Microbiology, University of Würzburg, Würzburg, Germany; 2 Institute of Mathematics, Chair of Mathematics VIII (Statistics), University of Würzburg, Würzburg, Germany; 3 Haartman Institute, Department of Virology, University of Helsinki, Helsinki, Finland; 4 Clinic of Venereal Diseases, Skin and Allergy Hospital, Helsinki University Central Hospital, Helsinki, Finland; 5 Department of Infectious Disease Surveillance and Control, National Institute of Health and Welfare, Helsinki, Finland; 6 Helsinki University Central Hospital, Laboratory Division (HUSLAB), Department of Virology and Immunology, Helsinki, Finland; Midwestern University, United States of America

## Abstract

Human herpesvirus-6 (HHV-6) exists in latent form either as a nuclear episome or integrated into human chromosomes in more than 90% of healthy individuals without causing clinical symptoms. Immunosuppression and stress conditions can reactivate HHV-6 replication, associated with clinical complications and even death. We have previously shown that co-infection of *Chlamydia trachomatis* and HHV-6 promotes chlamydial persistence and increases viral uptake in an *in vitro* cell culture model. Here we investigated *C. trachomatis*-induced HHV-6 activation in cell lines and fresh blood samples from patients having *Chromosomally integrated HHV-6* (CiHHV-6). We observed activation of latent HHV-6 DNA replication in CiHHV-6 cell lines and fresh blood cells without formation of viral particles. Interestingly, we detected HHV-6 DNA in blood as well as cervical swabs from *C. trachomatis*-infected women. Low virus titers correlated with high *C. trachomatis* load and vice versa, demonstrating a potentially significant interaction of these pathogens in blood cells and in the cervix of infected patients. Our data suggest a thus far underestimated interference of HHV-6 and *C. trachomatis* with a likely impact on the disease outcome as consequence of co-infection.

## Introduction

Human herpes virus 6 (HHV-6) is a ubiquitous pathogen with more than 90% seroprevalence in healthy adults. HHV-6 is commonly associated with self-limited childhood illness roseola infantum, and rarely with more severe syndromes. Chronic viral replication mostly occurs in salivary glands whereas HHV-6 persists in a latent stage in various tissue types including monocytes and bone marrow progenitor cells. Latent HHV-6 resides in the host cell as nuclear episome [1], [2] or integrated into telomeric ends of host cell chromosomes. The later form of latent HHV-6 can be vertically transmitted in the germ line [3], [4], [5]. Chromosomal integration of HHV-6 is described to be the most common form of latent HHV-6 [3]. After acquiring latency, HHV-6 persists in a dormant state with minimal viral transcription or translation in human host cells without any clinical complications. However, under various physiological conditions, latent HHV-6 is reactivated to form infectious viral particles (for further details see reviews [6], [7], [8]). In immune compromised patients, reactivation of viral activity may lead to severe limbic encephalitis [9]. HHV-6 reactivation has been correlated with relapsing and secondary progressive multiple sclerosis (MS) [10], [11], [12], [13] and Chronic Fatigue Syndrome (CFS) [11], [14]. Reactivation of latent HHV-6 can also be achieved by treatment with chemical stimulators like Trichostatin A (TSA), 12-O-tetradecanoyl-phorbol-13-acetate (TPA), calcium ionophore A23187 and hydrocortisone leading to viral replication in an extra-chromosomal state, viral gene transcription and formation of new infectious viral particles [3], [5], [15].

The Gram-negative, obligate intracellular bacterium *Chlamydia trachomatis* is the leading infectious cause of blindness and the third most frequent sexually transmitted infection worldwide. Epidemiological studies have connected HHVs and *Chlamydia* in several conditions. Herpes simplex virus 2 (HSV-2) is associated with *C. trachomatis* in endometritis and acute salpingitis [16]. HHV-6 DNA is frequently detected in different grades of cervical lesions [17], [18]. *C. trachomatis* infection has also been separately linked to cervical cancer [19] but it is unclear, whether both these pathogens contribute to the development of cervical cancer. *Chlamydia pneumoniae,* a highly prevalent pathogen with up to 80% serum antibody positivity in adults, is the cause of pneumonia in humans, but has also been associated with chronic diseases like atherosclerosis, progressive neurological disorders and lung cancer. In CFS patients, HHV-6 has been identified together with *C. pneumoniae* [20]. Although still controversially discussed, support for a role of *C. pneumoniae* and HHV-6 co-infection in the development and progression of MS has been provided [21], [22]. Although *C. trachomatis* and *C. pneumoniae* differ in their tissue tropism, they have a similar infection physiology. In a previous study, we have shown that co-infection with HHV-6 influences the development of infectious progeny of both, *C. trachomatis* and *C. pneumoniae* [23]. Moreover, different herpes viruses like herpes simplex virus (HSV)-1 and HSV-2 and human cytomegalovirus influence chlamydial infectivity in a similar way [23].

Here, we studied the influence of chlamydial infection on latent HHV-6 and its activation. Our data show that *Chlamydia* infection induces replication of chromosomally integrated HHV-6 in both immortalized human cells as well as in freshly isolated blood cells. To get a better insight into such interaction *in vivo*, we investigated cervical swabs from patients with *C. trachomatis*-infection for the HHV-6 and *C. trachomatis* load by qPCR. Our data provide statistically significant correlations supporting an interaction between HHV-6 and *C. trachomatis*.

## Materials and Methods

### Cell Lines, Patients and Samples

For the study of latent CiHHV-6 activation, established CiHHV-6 cell lines like HSB-ML (a tetraploid T-cell line derived from HBS-2 cells with 2–5 copies of CiHHV-6), JL-LCL and PL-LCL (cell lines established from CiHHV-6 individuals with 1–2 copies of HHV-6A per cell) were kindly provided by the HHV-6 foundation, USA (www.hhv-6foundation.org/, www.bioworldantibodies.com). Fresh blood samples from 5 individuals with CiHHV-6 were also provided by the HHV-6 foundation, USA. Viral load was re-verified by qPCR, which confirmed the CiHHV-6 status of these cells. Cervical swabs and blood samples were available from 73 female patients attending the outpatient Sexually Transmitted Infection (STI) clinic of Helsinki University Central Hospital, Helsinki, Finland. The patients were recruited to the study because of symptoms suggestive of *C. trachomatis* infection, or because notified by an infected partner. Cervical swabs were collected in 2 different sample buffers. Cervical swabs collected in Gen-Probe Aptima specimen collection media (Gen-Probe Inc., USA) were tested for *C. trachomatis* ribosomal RNA by Aptima Combo 2 Assay (Gen-Probe). The cervical samples collected in Copan UTM-RT media (Copan Diagnostics Inc., USA) were used for culture, then stored at -80°C and later used for total DNA extraction and detection of *C. trachomatis* and HHV-6 viral load. *C. trachomatis* antibodies were determined by micro-IF test.

HeLa cells containing latent HHV-6, HSB-2 and Molt-3 cells [23] were grown in RPMI 1640 media and 5% fetal bovine serum (FBS) at 37°C and 5% CO_2_.

### Ethics Statement

The CiHHV-6 blood samples were collected under written informed consent under IRB# CI001-HHV6 approved by The Essex Institutional Review Board Committee. Informed written consent was also obtained from all participants of the ChlamyTrans Study, and the ethics committee approved the consent procedure. A study permit was obtained from Helsinki University Central Hospital, Laboratory Division (HUSLAB). The Ethics Committee of the Department of Medicine, Hospital District of Helsinki and Uusimaa evaluated and approved the ChlamyTrans research plan (Dnro HUS 206/13/03/01/09, 19.08.2009). The Ethics Committee of the Department of Medicine (in Finnish: Sisätautien eettinen toimikunta), Hospital District of Helsinki and Uusimaa is an independent review board/ethics committee.

### Peripheral Blood Mononuclear Cell (PBMC) Isolation

Fresh PBMCs were first separated from whole blood using Histopaq1077 solution (Sigma, St. Louis, MO, USA) according to manufacturer’s instructions. Briefly, freshly collected blood was diluted 2 times with PBS and layered carefully onto 1 volume of Histopaq1077 solution without mixing the solutions. PBMCs were collected after centrifugation at 600×g for 30 min at room temperature. Cells were washed 3 times with 25 volumes of PBS and kept in RPMI 1640 media with 5% fetal bovine serum (FBS) at 37°C and 5% CO_2_. Isolated PBMCs were cultured on a plastic culture dish (NUNC, USA) for 48 h to separate monocytes, which later differentiate to macrophages. Separated adherent monocytes were further cultured for 5–7 days in presence of 50 ng/ml of macrophage colony stimulating factor (M-CSF) (Sigma, USA) to allow complete differentiation into macrophages. Both the adherent macrophage fraction and the PBMC fractions were infected with *C. trachomatis* separately.

### *Chlamydia Trachomatis* Infection

CiHHV-6 containing cells and PBMCs (both monocyte derived macrophages and leukocytes) were infected with *C. trachomatis* at an MOI of 5 either in an Eppendorf tube or on a culture plate and were centrifuged for 1 h at 37°C at 1200×g as described previously [23]. Cells were washed 3 times with PBS and grown in fresh RPMI 1640 media with 5% FBS.

### DNA Extraction from Cervical Swabs

Total DNA was extracted from the cervical swabs using MagNA Pure LC Total Nucleic Acid Isolation Kit (Roche Diagnostics, Germany) with minor modifications. Briefly, 1 ml of the cervical swab sample stored in Copan UTM-RT media was mixed with 100 µl of magnetic beads. To this, 450 µl of lysis/Binding binding buffer was added together with 150 µl of Proteinase-K and 1 µl of RNAse A. Samples were mixed thoroughly and incubated for 5 min at room temperature. Beads were separated by a magnet and subsequently 850 µl of wash buffer I was added. Beads were again separated and then washed with 450 µl of wash buffer II and then followed by another wash in 450 µl of wash buffer III. Bead bound DNA was eluted in 100 µl of elution buffer at 70°C. Removal of RNA from the DNA preparations was confirmed by analyzing random samples with DNAse treatment and reverse transcription PCR. Amount of DNA was quantified using NanoDrop 1000 Spectrophotometer (Thermo Scientific, Germany) and was stored at −20°C until further analysis.

### DNA Extraction From Blood

Total DNA was extracted from whole blood samples using QIAamp DNA Blood Mini Kit (Qiagen, Germany) following manufacturer’s protocol.

### Preparation of Plasmids for Standard Curve Analysis

HHV-6A (U1102), HHV-6B (Z29) [23], U94 ORF, *C. trachomatis* type III secretion chaperon LcrH/SycD, PI15 ORF were amplified using primer pairs described in Table 1. Amplified PCR products were agarose gel purified and were cloned into TOPO pCR 2.1 vector (Life Technologies, Germany). Cloned DNA vectors were transformed into *E. coli* DH5α and propagated to isolate ample amount of vectors for qPCR analysis. Cloned ORFs were verified by DNA sequencing.

**Table 1:** Detection of *Chlamydia* and HHV-6 in cervical swab samples and total blood.

*C. trachomatis*	HHV-6			
	Negative (n = 18)	Positive (n = 55)		
		HHV-6 positive (only in cervix)	HHV-6 positive (only in total blood)	HHV-6 positive (both in total blood and cervix)
Negative **(n = 25)**	**n = 7**	**n = 1**	**n = 5**	**n = 12**
Positive **(n = 48)**	**n = 11**	**n = 11**	**n = 13**	**n = 13**

73 cervical swab and total blood samples were tested by qPCR for *Chlamydia* and HHV-6.

### Quantitative Real time PCR (qPCR)

For qPCR, PerfeCTa™ qPCR SuperMix (Quanta Biosciences) was used and PCR amplifications were done on a StepOnePlus™ real time PCR platform (Applied Biosciences) using manufacturer’s protocol and SYBR® Green chemistry. qPCR reactions were performed using 100 ng of DNA per sample per well. Amplified data were analyzed using StepOne™ Software v2.1. Primer details are provided in table 1.

### Statistical Analysis

For categorically scaled measures, results are presented as numbers and frequencies. In case of metric data, mean, median and the standard deviation is given unless otherwise stated. To assess the relationship between two categorical measures, the chi-square test was used. When comparing a metric measure between two independent samples the normality assumption was checked first. If normality was given, the t-test was performed, otherwise the Wilcoxon test was used. For the comparison of more than two independent groups the Kruskal-Wallis test was conducted. The binomial test was done for the comparison of two proportions. All tests were two-tailed and differences were considered statistically significant if P<0.05. The software package used was IBM SPSS for Windows, version 19.0.

### Activation of HHV-6

To study HHV-6 reactivation, CiHHV-6 cell lines and fresh blood samples from individuals with CiHHV-6, were infected with *C. trachomatis* serovar L2 at an MOI of 1–5. Chlamydial infection was monitored by phase contrast microscopy. In parallel, CiHHV-6 cells were cultured in RPMI medium 1640 supplemented with 10% FCS together with 1×10^−6^ M hydrocortisone or 80 ng/ml Trichostatin A (TSA) (Sigma, USA) for 3–5 days, which are known to induce HHV-6 reactivation. After 2–3 days of infection, cells were grown in fresh media supplemented with 1 µg/ml of doxycycline, which allowed *Chlamydia* infected cells to recover. As HSB-2 and Molt-3 cells (T-cell derived cells) allows productive HHV-6 infection, 1×10^4^ HSB-2 or Molt-3 cells were added to 10^6^ PBMCs and co-cultured for 10–14 days in order to recover the reactivated viruses. Reactivation of HHV-6 was monitored by analysis of cytopathic effects, Gardella gel analysis and detection of viral genomes by qPCR.

### Gardella Gel Analysis

Gardella gel analysis allows the detection of large episomal DNA as it is formed as a consequence of HHV-6 reactivation. This method was used to separate episomal HHV-6 DNA from cellular DNA as described by Gardella et al. [24] with minor modifications. Briefly, 2.5×10^6^ CiHHV-6 cells co-cultured with HSB-2 or Molt-3 cells were directly lysed inside each well of a vertical polyacrylamide gel in a sample buffer containing 15% Ficol, 2 Kunitz units of RNAase type1A (Sigma, USA) per ml, and 0.01% bromophenol blue in TBE buffer and were layered with lysis buffer containing 5% Ficoll, 1% SDS, 1 mg of pronase (Sigma, USA) per ml and 0.05% xylene cyanol green. For lysis of isolated chlamydial elementary bodies (EB), samples were lysed in above mentioned lysis buffer containing lysozyme (Sigma, USA) at a final concentration of 7,500 U/ml. Gels were run at 4°C overnight and then stained in ethidium bromide for visualization of DNA. Subsequently, gels were used for southern transfer and hybridization.

### Southern Hybridization

Gardella gels were incubated in 0.125 M HCl for 10 min and then in DNA denaturation buffer (1.5 M NaCl, 0.5 M NaOH) for 30 min. DNA was transferred to Nylon-XL membrane (Amersham Hybond-XL, GE life sciences) by capillary transfer using denaturation buffer for transfer. After transfer, membrane was washed with neutralization buffer (3 M NaCl, 0.3 M Tri-sodium citrate, 0.5 M Tris, pH 8.0) for 15 min and was subsequently pre-incubated in hybridization buffer (GE life sciences, USA). After 1 h of pre-incubation, either random primed *C. trachomatis* probes (GE life sciences, USA) or end labeled HHV-6 oligo probes (Table S1) were added to the hybridization buffer and incubated overnight at 65°C or at 42°C, respectively. Membranes were washed and exposed overnight to phosphor storage screens (Fujifilm), which were then scanned by Typhoon 9200 imager (GE Healthcare). PCR amplified Ctr LcrH/SycD gene product of 136 bp was used for random priming and subsequent as probe.

### Neutral–neutral 2D DNA Electrophoresis

Separation of DNA in neutral–neutral 2D gels was performed as previously described [25]. Briefly, DNA was first separated on 0.4% agarose at low voltage in 0.5× TBE, and the gel was stained with 0.3 µg/ml ethidium bromide. The lane was cut and placed on a clean gel support at 90° to the direction of the first electrophoresis, cast with 1.1% agarose containing 0.3 µg/ml ethidium bromide and run in 0.5× TBE. The first dimension was run overnight at 1 V/cm, and the second at 4 V/cm for 3.5 h, both at room temperature. Southern blot analyses were performed as described above.

## Results

### *C. trachomatis* Infection Induces CiHHV-6 DNA Replication and Formation of Extra-chromosomal HHV-6 DNA

**Figure 1:**
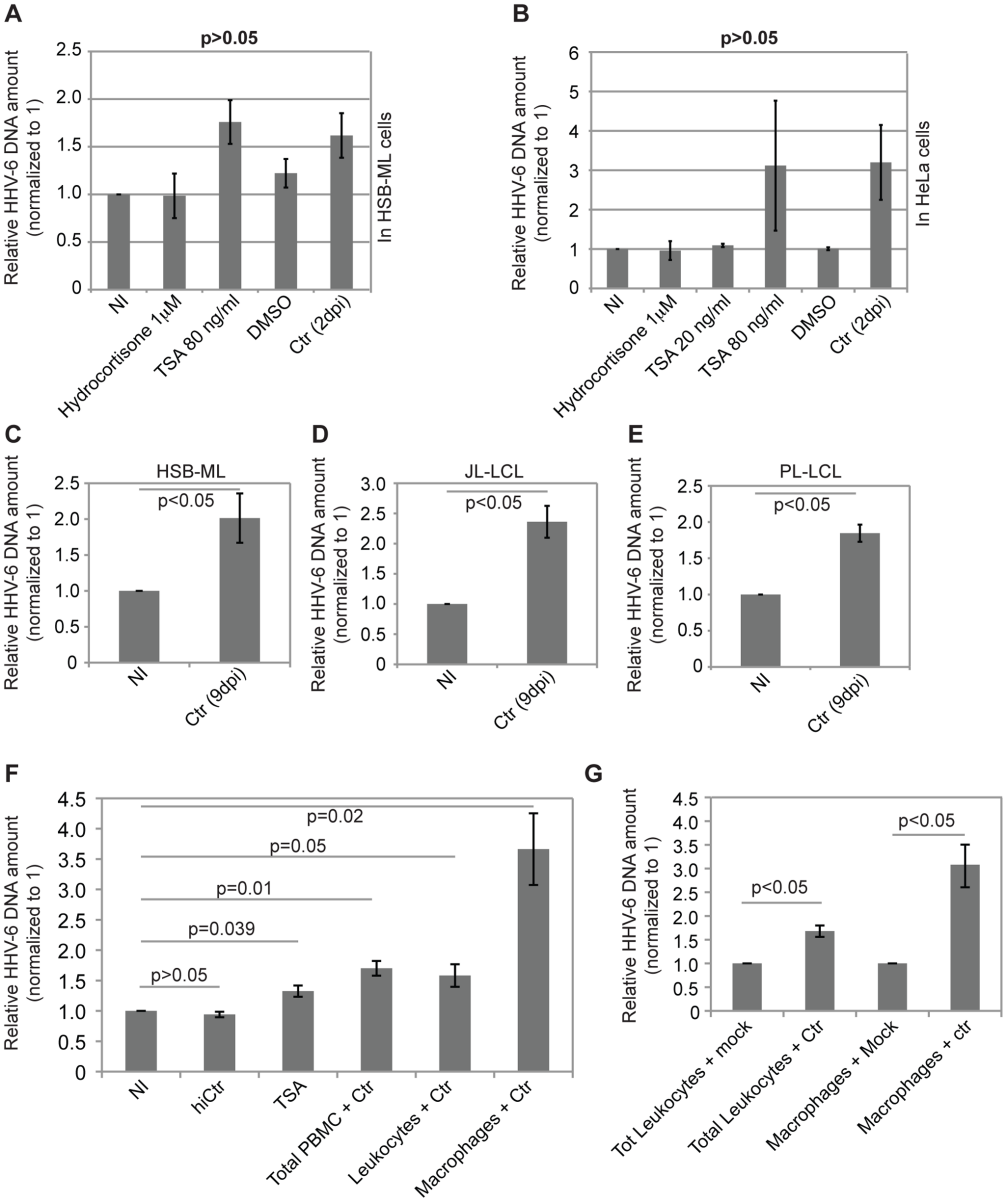
*Chlamydia trachomatis* infection induces CiHHV-6 DNA replication. (**A**) qPCR analysis of viral DNA in HSB-ML cells after 2 days of *C. trachomatis* infection. HSB-ML cells were infected with *C. trachomatis* (Ctr) for 2 days. In parallel, the same number of HSB-ML cells was cultured in the presence of 1 µM hydrocortisone or 80 ng/ml of TSA for 2 days. As TSA was dissolved in DMSO, a DMSO control was also included in the study. Data represent the mean ± SEM of 5 independent experiments. (**B**) qPCR analysis of viral DNA in HeLa cells carrying latent HHV-6A after 2 days of *C. trachomatis* infection. Cells were infected with *C. trachomatis* or were treated with TSA or hydrocortisone as mentioned in (A). Data represent the mean ± SEM of 5 independent experiments. (**C, D, E**) qPCR analysis of viral DNA in HSB-ML (C), JL-LCL (D) and PL-LCL (E) cells after 9 days of *C. trachomatis* infection. Cells were infected with *C. trachomatis* at an MOI of 2–5 for 2 days after which cells were washed thoroughly and grown in presence of fresh media with 1 µg/ml of doxycycline for another 7 days. Total DNA was isolated and used for qPCR assay. Data represent the mean ± SEM of three independent experiments. (**F**) qPCR analysis of viral DNA in freshly isolated blood cells from CiHHV-6 individuals after 5 days of *C. trachomatis* infection. Fresh PBMCs were isolated from total blood and were infected with *C. trachomatis* for 2 days at an MOI of 5 after which cells were washed thoroughly and grown in presence of fresh media with 1 µg/ml of doxycycline for another 3 days. In parallel, the same numbers of PBMCs were cultured in presence of 80 ng/ml of TSA for 5 days. Total leukocytes were separated from monocyte-derived macrophages and were infected with *C. trachomatis* in parallel. Heat inactivated *C. trachomatis* (hiCtr) were added to total PBMCs in a separate well and were included as negative control. Data represent the mean ± SEM of results from blood samples of 5 different CiHHV-6 individuals. (**G**) qPCR analysis of viral DNA in total leukocytes and monocyte-derived macrophages from CiHHV-6 individuals after 5 days of *C. trachomatis* infection. Total leukocytes were separated from monocyte-derived macrophages and were infected with *C. trachomatis* for 2 days at an MOI of 5 after which cells were washed thoroughly and grown in presence of fresh media with 1 µg/ml of doxycycline for another 3 days. Data represent the mean ± SEM of three independent experiments performed at the same time from one CiHHV-6 individual.

To test the effect of *C. trachomatis*-infection on latent chromosomally integrated HHV-6 (CiHHV-6) DNA replication, we infected HSB-ML cells carrying 1–2 copies of HHV-6A per cell with *C. trachomatis* (MOI 1) (Figure 1A). We also infected cervical epithelial HeLa cells carrying latent HHV-6A (100 copies of HHV-6A per 1000 cells) (Figure 1B) [23]. Total DNA extracted at 2 days post infection (dpi) and 9 dpi was used for quantitative PCR (qPCR) [23] against HHV-6 U94 ORF (Table S1). In parallel, all the cell lines were treated with hydrocortisone (1 µM) or TSA (80 ng/µl), which were previously shown to induce latent HHV-6 DNA replication [3]. DMSO solvent controls were included in separate wells/cultures and all experiments were repeated at least 5 times. We observed increased amount of viral DNA in both cell types at 2 days after *C. trachomatis* infection (Figure 1A, 1B). Hydrocortisone treatment did not induce any HHV-6 replication in our system. HHV-6 DNA replication induced by *C. trachomatis* infection was comparable to that observed upon treatment with the chemical inducers TSA. The increase in the amount of viral DNA, however, was not statistically significant because of high level of variation within the experiments. Interestingly, at 9 dpi the amount of viral DNA was significantly higher than in unstimulated cells (p<0.05) (Figure 1C). Similar experiments were carried out using 2 other CiHHV-6 cell lines (JL-LCL and PL-LCL), and a 1.8–2.4 fold increase in the amount of viral DNA at 9 dpi was observed (Figure 1D, 1E).

To test the effect of *C. trachomatis* on replication of latent HHV-6 in fresh PBMCs, we isolated PBMCs from 5 healthy individuals having CiHHV-6. The PBMC were either directly infected with *C. trachomatis* for 3 days or used for separation of monocytes from total leukocytes (see Material and Methods), which were further differentiated into macrophages. Total leukocytes and monocyte-derived macrophages were then separately infected with *C. trachomatis* (MOI 1–5) for 3 days. Heat inactivated *C. trachomatis* (hiCtr) was used as a (negative) control. Total DNA was extracted at 3 dpi and analyzed by qPCR. We observed a minor increase (1.3-fold) in the amount of viral DNA in the presence of TSA whereas a 1.7-fold increase was observed when total PBMCs were infected with *C. trachomatis* (Figure 1F). In order to check cell type specificity for *C. trachomatis* infection and subsequent viral activation, we analyzed the amount of viral DNA in total leukocytes as well as in monocyte-derived macrophages. We observed up to 3.6-fold increase in the amount of viral DNA in monocyte-derived macrophages whereas only a 1.6-fold increase was observed in total leukocytes. The increase in the amount of viral DNA in response to *C. trachomatis* infection was consistent in all the 5 samples tested and was statistically significant (p<0.05). Since not enough PBMCs from the five CiHHV-6 individuals were available to perform control experiments in non-infected total leukocytes and monocyte-derived macrophages, we repeated these important control experiments with PBMCs from one CiHHV-6 individual (Figure 1G) and thereby corroborated the results shown in figure 1F.

### Increased Viral DNA Replication is not Sufficient to Produce Infectious Viral Particles

**Figure 2:**
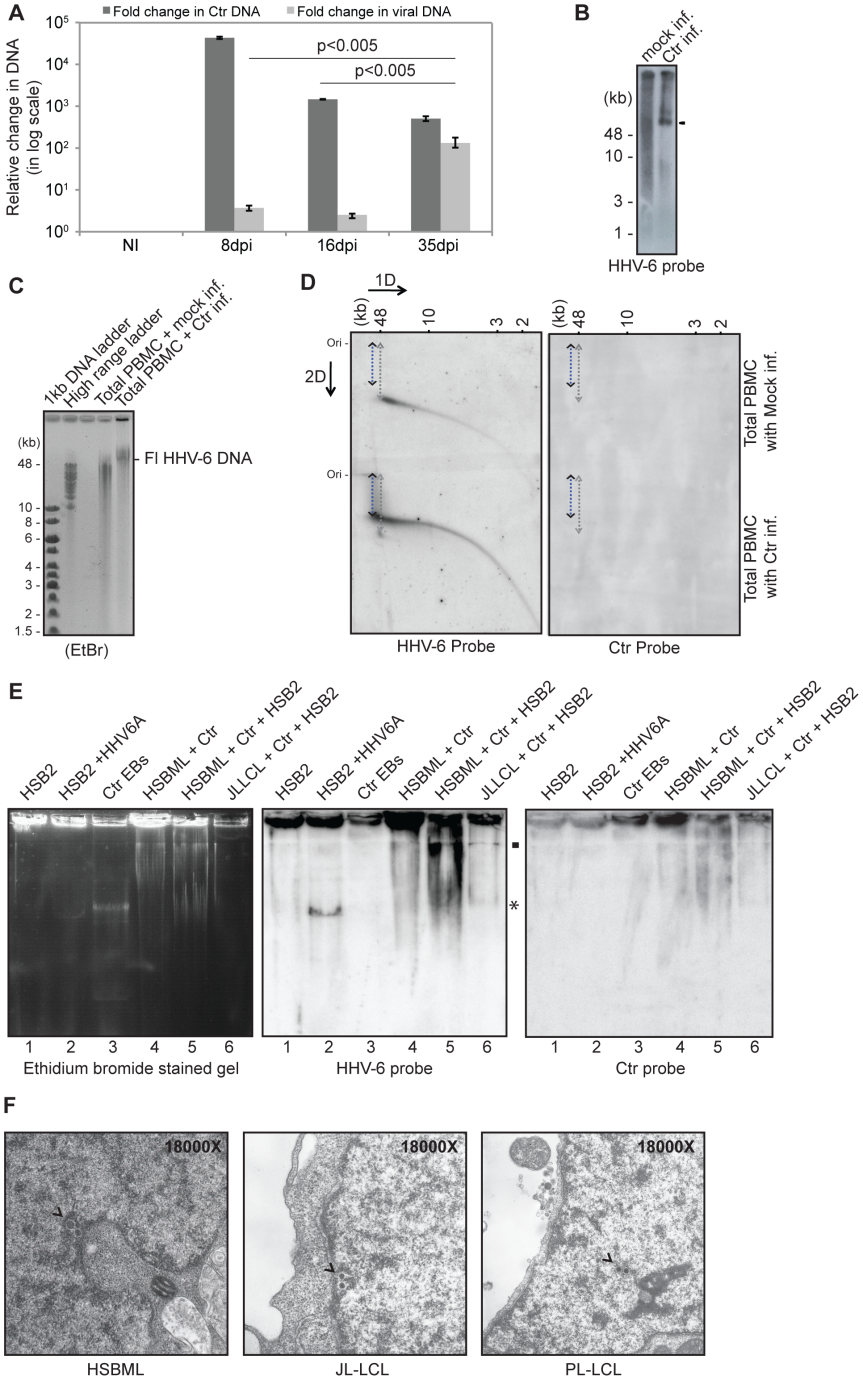
*C. trachomatis* infection induces formation of extra-chromosomal HHV-6 DNA in CiHHV-6 cell lines and patient blood samples. (**A**) *C. trachomatis* infection induces viral DNA replication in HSB-ML cells. HSB-ML cells were infected with *C. trachomatis* at an MOI of 2 for 2 days after which cells were washed thoroughly and grown in presence of fresh media with 1 µg/ml of doxycycline for 35 days. Total DNA was isolated at 3 different time intervals and used for qPCR assay. Data represent the mean ± SEM of 3 independent experiments. (**B**) Southern hybridization of *C. trachomatis*-infected HSB-ML cells shows detectable amount of extra-chromosomal HHV-6 after 35 days of infection. Total DNA from non-infected (NI) and 35 days post infected (dpi) HSB-ML cells were run on 1% agarose gel and were subsequently used for southern transfer and hybridization. HHV-6 specific band is marked by dark arrowhead. (**C**) Ethidium bromide (EtBr) stained agarose gel showing full-length HHV-6 DNA in total PBMCs of a CiHHV-6 individual after 16 days of Chlamydia treatment. Fl, full-length. (**D**) Same gel as in (C) was processed for neutral-neutral 2D DNA electrophoresis and subsequent hybridization with a HHV6 probe and then with *C. trachomatis* probe. Migration rate of full-length HHV-6 DNA from *C. trachomatis*-infected cells is different from that of mock infected cells. Ori, the position of the origin of the second-dimension gel electrophoresis. The positions of the size markers for the first-dimension gel electrophoresis are shown at the top. (**E**) Gardella gel analysis and subsequent southern hybridization showing extra-chromosomal HHV-6 DNA after *C. trachomatis* infection. HSB-ML and JL-LCL cells were infected with *C. trachomatis* (MOI 5) for 2 days after which cells were washed thoroughly and grown in presence of fresh media with 1 µg/ml of doxycycline for another 7 days. Fresh HSB-2 cells were added to these cells and were co-cultured for another 2 weeks. 2.5×10^6^ of these cells were loaded directly onto a Gardella gel and subsequently stained with ethidium bromide and afterwards transferred to a nylon membrane. HSB-2, HHV-6-infected HSB-2 for productive virus infection (HSB-2+HHV-6), *C. trachomatis* elementary bodies (Ctr EB) and *C. trachomatis*-infected HSB-ML cells, which were subsequently not co-cultured with HSB-2 cells (HSB-ML+Ctr) were loaded as controls. Nylon membrane was hybridized first with a HHV-6-specific probe and subsequently stripped and used again for hybridization with *C. trachomatis*-specific probe. Extra-chromosomal linear HHV-6 DNA is marked with *and possible nicked circular viral DNA is marked with a rectangle. Chlamydial DNA did not run in the gel due to the size of genomic DNA and hence *C. trachomatis*-specific hybridization was observed only in the wells of lane 4 and 5. A clear band (probably the chlamydial plasmid) in lane 3, which was observed after ethidium bromide staining was not detected by *C. trachomatis*-specific probe. Instead some additional bands were detected in lane 4 with *C. trachomatis*-specific probes. (**F**) Transmission electron microscopy pictures showing appearance of particle like condensed DNA structures (marked by black arrowhead) in CiHHV-6 cell nuclei after *Chlamydia* infection.

To test the long-term influence of *C. trachomatis* infection on HHV-6 DNA replication, we repeated infections in HSB-ML cells and kept the cells in culture till 35 days in presence of 1 µg/ml of Doxycycline. The amount of viral DNA was increased up to 173-fold within 35 days of first *C. trachomatis* infection (Figure 2A), suggesting a strong induction of HHV-6 DNA replication under these conditions. Southern hybridization of total DNA from these cells verified the replication and presence of extra-chromosomal HHV-6 DNA in 35 dpi HSB-ML cells, which represents the activated HHV-6 DNA (Figure 2B). We repeated this experiment in fresh PBMCs from one of the CiHHV-6 individuals and observed appearance of full-length extra chromosomal HHV-6 DNA (Figure 2C) after 16 days of *C. trachomatis* infection. Further long-term culture was not possible with isolated PBMCs because of their limited life span in cell culture. We detected small viral DNA (smaller than 48 kb) in mock-infected PBMCs without having any full-length HHV-6 DNA. To differentiate extra-chromosomal HHV-6 DNA between mock-infected and *C. trachomatis*-infected PBMCs, we performed neutral-neutral 2D DNA electrophoresis (Figure 2D), which differentiates DNA in 2 dimensions, by mass and by conformation. We observed clear differences between both the DNAs thus confirming our hypothesis that *C. trachomatis* infection induces CiHHV-6 DNA replication and formation of extra-chromosomal HHV-6 DNA. Hence our results show that *C. trachomatis* infection promotes formation of extra-chromosomal HHV-6 DNA and its replication in various cell types.

It has been previously shown that chemically induced latent HHV-6 activation may or may not lead to formation of new viral particles [3], [26], [27], [28]. To test whether *C. trachomatis*-induced HHV-6 DNA replication results in the formation of new infectious viral particles, latently infected JL-LCL or HSB-ML cells were co-cultivated with HSB-2 cells, which are permissive for the production of HHV-6 viral particles. HSB-ML and JL-LCL were infected with *C. trachomatis* (MOI 1–5) for 2–3 days, and then grown in doxycycline (1 µg/ml) containing media for additional 7 days to avoid lysis of the cells due to chlamydial replication. These cells were then co-cultured with fresh HSB-2 cells in order to allow HHV-6 infection and propagation. After 2 weeks of co-culture, 2.5×10^6^ cells were collected and used for Gardella gel analysis (to look for extra-chromosomal viral genomes). The co-culture experiments were repeated at least 3 times. We detected low amounts of both linear as well as circular HHV-6 DNA molecules in all experiments (Figure 2E, S1). However, we could not detect any infectious viral particles in the co-cultured cells by transmission electron microscopy (data not shown). Interestingly, we observed slow migrating, slightly higher molecular weight HHV-6 extra-chromosomal DNA in co-cultured HSB-ML and JL-LCL cells pointing to the possibility of additional confirmations for HHV-6 DNA or presence of additional viral or non-viral sequences than the known linear double stranded DNA (Figure 2E, S1). In addition, we observed either nicked circular or concatemeric HHV-6 DNA in all the co-cultured cells (Figure 2E, S1), suggesting circularization of HHV-6 DNA. In line with these observations, electron microscopic analysis of *C. trachomatis*-infected HSB-ML, JL-LCL and PL-LCL cells could not show presence of any viral particles in these cells. Only condensed particle like DNA structures were detected in their nuclei (Figure 2F). Thus, our data shows that *C. trachomatis* infection reactivates replication of HHV-6 in CiHHV-6 cells without leading to infectious virus particle formation.

### qPCR Assays for Quantification of HHV-6 and *C. trachomatis* DNA

Our previous [23] as well as the present study using cell culture models indicated that *C. trachomatis* infection has an effect on active as well as latent HHV-6 infection. To investigate the nature of *C. trachomatis* and HHV-6 co-infection in a clinically relevant situation, we established methods to detect these microbes in patient samples. Latent HHV-6 DNA can be detected in various body parts [29], [30] including cervical epithelial tissues [31], [32] where they influence human papillomavirus (HPV) gene regulation [2]. *C. trachomatis* is a mucosal pathogen and its DNA can be detected in various grades of cervical neoplasia as well as in asymptomatic patients [33], [34]. Hence, the possibility of an interaction between *Chlamydia* and HHV-6 in cervical epithelial cells is plausible.

We therefore studied cervical swabs from 73 female patients and determined *C. trachomatis* load and HHV-6 viral load. As the number of cervical epithelial cells in such samples can be quite low, we developed a highly efficient SYBR green- based quantitative real time PCR (qPCR) assay to detect low amounts of microbial DNA in small samples.

**Figure 3:**
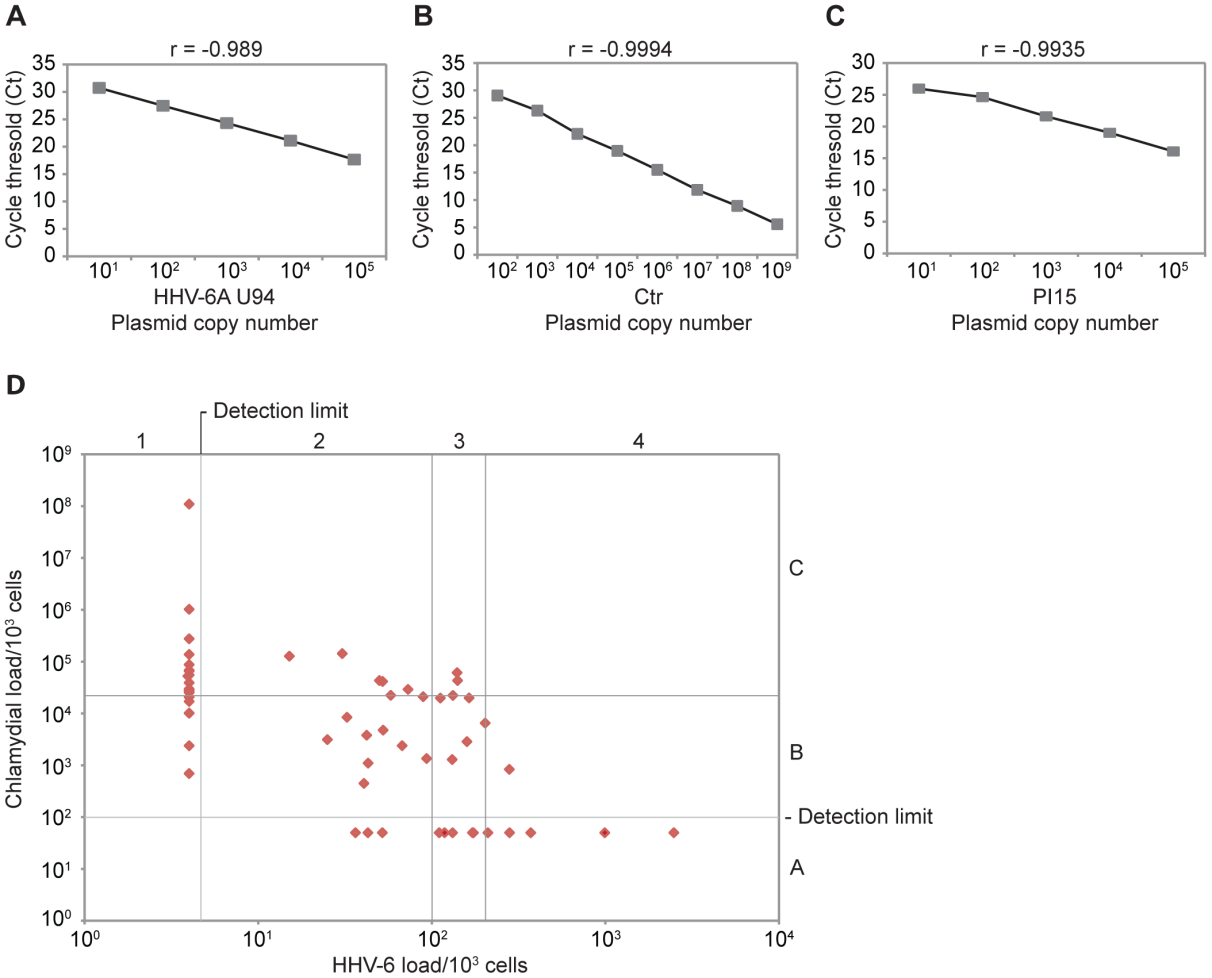
Quantitative real time PCR assay to detect HHV-6 and chlamydial load in cervical swabs and total blood samples. (**A–C**) Standard curves for HHV-6 and *C. trachomatis* quantification. Ten fold serial dilutions of U94 HHV-6A (**A**), *C. trachomatis* (**B**) and PI15 (**C**) plasmids were tested in triplicates in qPCR reaction. Mean Ct values were plotted against the copy numbers. Correlation coefficients and slope values are mentioned in each figure. (**D**) Scatter plot showing quantitative distribution of HHV-6 and *C. trachomatis* in cervical swabs of patients (n = 65) with suspected *C. trachomatis* infection as determined by real-time qPCR assay. Samples with no detectable HHV-6 and *C. trachomatis* are not included. Samples with HHV-6 DNA below detection limit (5 copies/10^3^ cells) were arbitrarily specified as 4 copies/10^3^ cells. Similarly, samples with less than 100 copies/10^3^ cells were arbitrarily specified as 50 copies/10^3^ cells. Sensitivity of PCR was marked as detection limits.

For preparation of standard curves, we cloned a PCR-amplified full-length HHV-6A (U1102 strain), HHV-6B (Z29) U94 (1473 bp) ORF and a 136 bp fragment of *C. trachomatis* type III secretion chaperon LcrH/SycD into TOPO pCR 2.1 (Life Technologies, Germany) vector backbone. Primers for HHV-6 U94 ORF, one of the well-studied transcripts essential for HHV-6 latency, were selected for qPCR and standard curve analysis [23], [35]. As the type III secretion chaperon of *C. trachomatis* is essential for chlamydial survival and infection, it was selected for qPCR as well as standard curve analysis. For normalization of microbial DNA to cellular DNA, we selected PI15 ORF (NM_015886), a gene that exists as a single copy per haploid genome (or 2 copies per human cell) and cloned the full-length PI15 (777 bp) into the same vector backbone. The recombinant plasmid standards were diluted serially 10-fold starting from 1 plasmid copy per µl to 10^6^ copies per µl. The standards were then amplified in triplicates (1–10^6^ copies per well) in a single run and the data collected were used to generate a linear-log regression plot. To evaluate inter- and intra-experimental variation, we repeated these tests at least 5 times and for 5 different time points and calculated the co-efficient of variation (CV) (Table S2). For all the 3 standards, mean Ct values from triplicates were plotted against plasmid copy number per reaction, which resulted in a linear graph (Figure 3A–C). The calculated detection limit was 5 copies for HHV-6, 100 copies for *C. trachomatis* and 100 copies for PI15 per reaction with a CV value of less than 5%. Detection of HHV-6, *C. trachomatis* and PI15 was highly efficient over a wide range (5 to 10,000 copies per reaction). Ten randomly selected PCR products from each PCR were verified by sequencing.

### HHV-6 and *C. trachomatis* Load in Cervical Swabs and Total Blood DNA

The established qPCR assays were utilized to detect HHV-6 and chlamydial DNA in cervical swabs as well as in DNA of total blood of patients. Based on qPCR results, the distribution pattern of different patient samples is shown in Table 1. A scatter plot was generated to visualize the correlation of HHV-6 and chlamydial load in cervical swab samples (only samples, which were either positive for HHV-6 or *Chlamydia* or for both (n = 65) were included) (Figure 3D).

Interestingly, we observed 2 clearly different groups of patients in terms of the viral and chlamydial load in their cervical swabs. The *C. trachomatis* negative group (n = 22) comprised patients with symptoms suggestive of genital infection or with partners having *C. trachomatis* infection, but negative serology as well as negative *C. trachomatis* NAAT test (Table 1, 2). We could detect chlamydial DNA in cervical samples from two of these patients, whereas 20 were negative also by our qPCR assay. Interestingly, 18 out of these 22 patients had high viral load either in blood or in cervical swabs or both. 12 out of these 18 HHV-6 positive patients had detectable HHV-6 in cervical swab samples and all of them had higher viral load in cervical swabs than their blood.

**Table 2:** HHV-6 and chlamydial load in the *Chlamydia* negative control group.

	Sample	Ctr IgG	Ctr IgM	Ctr IgA	NAAT results	Blood virusload/10^3^ cells	Cervical virusload/10^3^ cells	Cervical Ctrload/10^3^ cells
1	CT103	<32	ND	ND	NEG	7.012237438	96.5	ND
2	CT104	<32	ND	ND	NEG	11.48199855	42.855	ND
3	CT105	<32	ND	ND	NEG	2.636135549	ND	ND
4	CT108	<32	ND	ND	NEG	15.47424588	ND	ND
5	CT114	<32	ND	ND	NEG	3.039837995	173.5656	ND
6	CT115	<32	ND	ND	NEG	8.717799266	1012	ND
7	CT116	<32	ND	ND	NEG	14.84461638	ND	ND
8	CT117	<32	ND	ND	NEG	32.5336175	486	ND
9	CT118	<32	ND	ND	NEG	7.909478419	51.8807	ND
10	CT 121	<32	ND	ND	NEG	20.55072928	280.3494	ND
11	CT123	<32	ND	ND	NEG	10.54691423	110.25	ND
12	CT134	<32	ND	ND	NEG	0.749681205	2479.25	ND
13	CT140	<32	ND	ND	NEG	5.858719662	ND	ND
14	CT143	<32	ND	ND	NEG	ND	ND	ND
15	CT146	<32	ND	ND	NEG	ND	210.4035	ND
16	CT148	<32	ND	ND	NEG	3.338386225	ND	ND
17	CT149	<32	ND	ND	NEG	ND	ND	ND
18	CT154	<32	ND	ND	NEG	ND	ND	ND
19	CT157	<32	ND	ND	NEG	6.119869271	371.481	ND
20	CT162	<32	ND	ND	NEG	ND	ND	ND
21	CT177	<32	ND	ND	NEG	8.629805364	140.6814	43184.9312
22	CT179	<32	ND	ND	NEG	11.41451537	ND	25399.2733

Samples with negative NAAT results were grouped in this table. HHV-6 DNA load in cervical smears as detected in qPCR assays is shown together with the HHV-6 DNA load in total blood from the respective samples. Serological data for *Chlamydia* (Ctr) IgG, IgM and IgA has also been included. Ctr IgG values less than 32 (<32) indicate IgG negative. ND, not detected; NEG, negative test result.

Another group (n = 21) of patients were positive for *C. trachomatis* DNA but no HHV-6 DNA was detectable (Table 1, 3) in their respective cervical swabs. 18 patients had also serological evidence of *C. trachomatis* infection: 8 had IgM, 16 had IgG and four had both IgG and IgM antibody. In 13 of the 21 patients HHV-6 DNA was detected in the blood.

**Table 3:** Patients with no detectable HHV-6 in cervical smears.

	Sample	Ctr IgG	Ctr IgM	Ctr IgA	Cervical virus load/10^3^ cells	Cervical Ctr load/10^3^ cells	Blood virus load/10^3^ cells
1	CT100	<32	ND	ND	ND (<5)	275809.309	7.99
2	CT124	128	ND	ND	ND (<5)	17020.3654	21.94766605
3	CT127	<32+/−	ND	ND	ND (<5)	28133.5987	6.04619509
4	CT129	128	ND	ND	ND (<5)	20731.6709	6.483198133
5	CT133	64	80	ND	ND (<5)	55086.4249	13.87471468
6	CT39	<32	40	ND	ND (<5)	137903.563	10.05636142
7	CT180	512	ND	40	ND (<5)	26844.5309	3.069298963
8	CT202	<32	320	ND	ND (<5)	29705.482	ND
9	CT219	<32	320	40	ND (<5)	109665172	ND
10	CT194	<32	160	ND	ND (<5)	52664.978	ND
11	CT159	64	ND	ND	ND (<5)	2382.5124	ND
12	CT150	128	ND	ND	ND (<5)	65655.5437	ND
13	CT152	<32	ND	ND	ND (<5)	1024506.99	ND
14	CT155	<32+/−	160	20	ND (<5)	10184.9924	ND
15	CT179	<32	ND	ND	ND (<5)	25399.2733	11.414
16	CT166	256	80	ND	ND (<5)	39040.4813	8.052790338
17	CT172	128	ND	ND	ND (<5)	28609.4354	13.46552309
18	CT158	<32+/−	ND	ND	ND (<5)	87336.546	1.606838633
19	CT144	128	40	ND	ND (<5)	68366.6735	5.0838
20	CT142	32	ND	ND	ND (<5)	10114.0679	ND
21	CT220	512	ND	ND	ND (<5)	693.45099	1.193656128

HHV-6 DNA load in cervical smears as detected by qPCR assay is shown together with the HHV-6 DNA load in total blood from respective samples. Serological data for *Chlamydia* (Ctr) IgG, IgM and IgA has also been included. Ctr IgG values less than 32 (<32) indicate IgG negative. ND, not detected.

We detected both HHV-6 and *C. trachomatis* DNA in cervical swabs of 23 patients (Table 1, 4). In 12 of these 23 patients, higher levels of viral DNA could be detected in cervical swab samples than in the blood. In the blood of the remaining 11 patients, HHV-6 DNA was not measurable. All these patients had detectable chlamydial DNA in the same cervical sample. 20 of these patients (90.9%) were positive for *C. trachomatis* IgG antibody indicating previous exposure to *C. trachomatis* or long-term infection, and 6 of them had also IgM antibody. Interestingly, none of these patients had high chlamydial load in their cervical swabs. In conclusion, our qPCR assay detected high HHV-6 load in cervical swab samples from patients with *C. trachomatis* infection supporting an interaction between these two microbes also in patients.

**Table 4:** Co-existence of HHV-6 and *Chlamydia* in cervical smears.

	Sample	Blood virusload/10^3^ cells	Cervical virusload/10^3^ cells	Cervical Ctrload/10^3^ cells	Ctr IgG	Ctr IgM	Ctr IgA	NAAT test
1	CT101	24.020291	67.4347	2387.141	128	ND	ND	POS
2	CT106	20.200325	112.0317	19837.0988	128	ND	ND	POS
3	CT107	37.025921	139.8426	61211.732	64	40	ND	POS
4	CT109	13.846686	72.9132	29160.0152	64	<20+/−	ND	POS
5	CT110	61.280771	93.32419	1349.38604	128	ND	ND	POS
6	CT128	0.481486	52.1045	41414.9428	512	160	20	POS
7	CT184	ND	159.3826	2865.9608	128	ND	ND	POS
8	CT185	8.900063	164.0614	19937.801	256	ND	ND	POS
9	CT213	ND	279.5814	835.9657	128	ND	ND	POS
10	CT214	ND	40.6256	447.44	32	ND	ND	POS
11	CT208	ND	42.961	1099.5269	256	ND	ND	POS
12	CT204	ND	89.1299	21083.946	64	ND	ND	POS
13	CT191	ND	132.3496	22272.932	64	ND	ND	POS
14	CT187	ND	130.9461	1291.9818	32	ND	ND	POS
15	CT203	ND	30.4866	143097.31	<32+/−	ND	ND	POS
16	CT210	89.257277	42.15	3811.1949	64	ND	ND	POS
17	CT153	ND	15.12106	127449	<32	160	ND	POS
18	CT163	ND	49.8583	43224.5877	64	ND	ND	POS
19	CT174	7.129155	32.4432	8443.53909	64	ND	ND	NEG
20	CT178	13.465523	52.4139	4763.27749	32	160	ND	POS
21	CT176	3.801527	57.9939	22412.0638	<32+/−	20	ND	NEG
22	CT175	10.633452	25.0302	3125.70194	<32+/−	ND	ND	POS
23	CT206	ND	203.0779	6519.2924	–	–	–	POS

Samples with detectable HHV-6 DNA as well as chlamydial (Ctr) DNA in cervical smears were grouped in this table. HHV-6 DNA load in cervical smears and in total blood from the respective samples are shown together with serological data for *Chlamydia* IgG, IgM and IgA. Ctr IgG values less than 32 (<32) indicate IgG negative. ND, not detected; POS, positive test result; NEG, negative test result.

## Discussion

*C. trachomatis* is a sexually transmitted mucosal pathogen and very frequently detected in cervical epithelial cells of infected patients [36], [37]. Yet, role of chlamydial infection in these patients is not well understood. In our previous report, we showed that active HHV-6 infection induces chlamydial persistence in HeLa cells. On the other hand, *Chlamydia* infection favors HHV-6 infection and survival. As most humans acquire HHV-6 infection early in life and *C. trachomatis* infection later, we addressed the question whether *C. trachomatis* infection can activate latent HHV-6 infection. Several chemical stimuli have been shown to activate viral replication in CiHHV-6 cell lines leading to formation of infectious viral particles [3]. Therefore, we tested 3 CiHHV-6 cell lines as well as PBMCs from 5 individuals having CiHHV-6 for their ability to induce viral replication after *C. trachomatis* infection. We observed an increase in the amount of viral DNA in all the cell types within 5–9 days after *C. trachomatis* infection. This increase in viral DNA was comparable to that induced by known chemical inducers like TSA [3]. However, unlike reactivation with chemical stimuli, long-term co-culture experiments did not result in infectious virus production. It is currently unclear, why formation of infectious viral particles was not detected after long-term culture. While chemical inducers are easily accessible for every cell in a particular experiment, *Chlamydia* infection is highly asynchronous in cultured suspension cells. Under most successful infection conditions, only 40–70% of the cells can be infected with *C. trachomatis*. Also, *C. trachomatis* has a highly lytic life cycle in cultured cells, which prevents development of long term *Chlamydia* infection *in vitro*. Thus, it is possible that any signal for HHV-6 activation resulting from *Chlamydia* infection is only available for limited time *in vitro*, preventing complete activation of HHV-6 DNA replication in our co-culture experiments.

Activation of HHV-6 leading to extra-chromosomal HHV-6 DNA formation might be the initial stage for virus activation. We observed formation of extra-chromosomal linear HHV-6 DNA in all the CiHHV-6 cell lines after *C. trachomatis* infection as well as in total PBMCs from one of the CiHHV-6 individuals. We detected HHV-6 DNA smaller than 48–kb in length in mock infected PBMCs from the CiHHV-6 individual (Figure 2C, 2D), which might represent spontaneously activated and incomplete HHV-6 DNA in these cells or can originate from community-acquired HHV-6 strains. In addition, we detected slowly migrating HHV-6 DNA in *C. trachomatis* infected CiHHV-6 cells co-cultured with permissive HSB-2 cells (Figure 2E, S1), which might represent either nicked circular or concatemeric HHV-6 DNA. In both cases, generation of extra-chromosomal circular HHV-6 DNA can be postulated as reason for this observation. Circular HHV-6 DNA is rarely detected [1], [2] in some of the cell lines infected with HHV-6, but the exact role of such circular viral DNA is unclear. Circular viral genome is postulated to be the start of replication for different herpes viruses, which can form precursors for linear viral DNA by rolling circle amplification mechanism [38], [39], [40], [41], [42]. Thus our results suggest possible viral activation in these cells in response to *C. trachomatis* infection, which leads to formation of extra-chromosomal HHV-6 DNA.

To check whether HHV-6 activation is restricted to certain cell types, we infected total blood leukocytes and monocyte-derived macrophages with *C. trachomatis*. Although we observed increase in the amount of viral DNA in both the cell types, HHV-6 DNA replication was more efficient in human macrophages. This may be due to the fact that macrophages are more susceptible to *C. trachomatis* infection than in leukocytes [23] thus leading to a better induction signal for HHV-6 DNA replication. It will be interesting in future to see the effect of increased viral DNA accumulation on transcription and translation profile of HHV-6 and its consequence on chlamydial growth.

To obtain evidence for a role of *C. trachomatis* and HHV-6 infections in clinical settings, we analyzed cervical swabs taken from patients with suspected *C. trachomatis* infection for the presence of HHV-6 and chlamydial DNA by qPCR. Results obtained with our established *C. trachomatis* qPCR assay correlated very well with those obtained using a commercial NAAT test with 91.7% of the specimens having similar results by both detection methods. The few discrepant results may be due to the sample collection as separate cervical swabs collected in different media were used for NAAT and the qPCR assay (see Material and Methods), or different analytical sensitivities (rRNA detection vs. DNA detection). Besides being qualitative, our qPCR assay could also quantitate the *C. trachomatis* load. We detected HHV-6 DNA in 48% of the tested samples. Although the amount of HHV-6 DNA in cervical swabs of *Chlamydia*-infected patients is comparatively high, HHV-6 or other HHVs have been frequently detected in cervical swabs earlier [17], [43], [44], [45]. We also detected HHV-6 DNA in blood cells of 59% of the patients. Although HHV-6 DNA has previously been detected in PBMCs of 40–90% of healthy individuals [46], the viral DNA copy numbers have not been determined in these studies. Thus, the detection of 10^3^–10^4^ copies of HHV-6 DNA per 10^6^ cells in total blood of *C. trachomatis*-infected patients points to a high probability of viral activation in all these patients.

We observed high viral loads either in total blood or cervical swabs from 55 patients. Interestingly, 81.8% of the patients in the *C. trachomatis* negative group had activated HHV-6 in their blood. HHV-6 levels in the whole blood of healthy individuals range between 5–10 copies per ml of blood [30]. Similarly, in CiHHV-6 patients, HHV-6 DNA levels exceed 5.5 log10 copies/ml [4]. HHV-6 DNA load in total blood, which lies in between these two extremes, indicates an activated state of the virus. Remarkably, 66.7% of the *C. trachomatis* negative patients with activated HHV-6 in their blood also had high viral titers in cervical epithelial cells ranging up to 5–6 log10 per 10^6^ cells in 10 of the samples (Table 2). Cervical epithelial cells are not known to be permissive for productive HHV-6 infection. High viral DNA in these cells could also come from blood cells derived from blood vessels associated with cervical tissue. But this assumption may not be valid as the viral titer was comparatively low in whole blood samples in the respective patients. Thus, we assume that high viral titer in cervical swabs could originate from localized activation of viral replication in cervical epithelial cells or in blood cells that enrich cervical epithelium and thus come in contact with cervical epithelial cells.

We observed coexistence of HHV-6 and *Chlamydia* in 23 samples (Table 4). Viral load was high in all these samples indicating HHV-6 activation. Similar to the previous observation, the blood HHV-6 load was comparatively low or negative in all these 23 patients in comparison to the viral load in their cervix. This could again be due to localized activation of HHV-6 in cervical epithelial cells in the presence of *Chlamydia* infection. Presence of chlamydial IgG antibody in 21 of these patients indicates the presence of chronic or long-term *C. trachomatis* infection, which supports our results from cell culture based HHV-6 reactivation studies.

For a better understanding of our study results, we divided the patients into 4 subgroups according to the viral load (group 1, 2, 3 and 4) and according to the chlamydial load into 3 subgroups (group A, B and C) (Figure 3D). Using the Kruskal-Wallis test, we observed a negative correlation between viral and bacterial load (Table S3). However, due to high degree of standard deviation, particularly within the samples with high chlamydial DNA load the correlation did not reach statistical significance. Low chlamydial DNA load has been detected in incident *C. trachomatis* infections in comparison to prevalent infections [36]. This could indicate that an active chlamydial infection might be associated with high chlamydial load whereas persistent or recovered infections would have lower organism loads. Thus, we excluded the samples (n = 22) with high chlamydial load (group A) and analyzed the patients with no *C. trachomatis* and low to moderate *C. trachomatis* load (group B and C). A statistically significant association between chlamydial and viral load was observed in these subgroups (Table S4). A two-way chi-square test within these subgroups also showed statistically significant association (Table S5).

In contrast to Kruskal-Wallis ANOVA test involving total samples, a two-way chi-square test revealed a statistically significant negative association (p<0.05) between viral and bacterial load (Table 5). Samples with high viral titer in cervix had a low chlamydial load whereas samples with low viral titer in cervix had a high chlamydial load (Figure 3D).

**Table 5:** Two-way Chi-square test to study the association between *Chlamydia* and HHV-6 load in cervical smear of all patients.

Chlamydial load**	HHV-6 viral load (per 10^3^ cells)*				
	Group 1 (<5)	Group 2 (5–100)	Group 3 (100–200)	Group 4 (>200)	Total no. of samples
No. of samples within group A	15	5	2	0	22
Rel. frequency within group A	68,2%	22,7%	9,1%	0,0%	100,0%
No. of samples within group B	6	10	5	2	23
Rel. frequency within group B	26,1%	43,5%	21,7%	8,7%	100,0%
No. of samples within group C	17	3	4	4	28
Rel. frequency within group C	60,7%	10,7%	14,3%	14,3%	100,0%
Total no. of samples	38	18	11	6	73
Rel. frequency within all groups	52,1%	24,7%	15,1%	8,2%	100,0%

* Samples have been arbitrarily divided into 4 sub groups (group 1, 2, 3 and 4) depending on the HHV-6 viral load. Respective HHV-6 DNA load is mentioned within brackets.

** Similarly, all the samples have been arbitrarily divided into 3 sub groups (group A, B and C) depending on the chlamydial (Ctr) DNA load. Group A: more than 25,000 copies of Ctr/1000 cells, Group B: between 100–25,000 copies/1000 cells, Group C: Below 100 copies of Ctr (not detectable)/1000 cells.

We observed 16 cases where both cervical swab as well as total blood was negative for HHV-6 DNA. In comparison, 22 samples had high viral load in blood whereas no HHV-6 was detected in their respective cervical swabs. As we cannot confirm the source of viral activation in whole blood of these 22 patients, we analyzed the rest of the 35 cases where high HHV-6 DNA titer was observed in cervical swabs (n = 34) in comparison to their whole blood counterpart by a binomial test (Table S6). We observed a statistically significant preference for viral activation in cervical epithelial tissues compared to whole blood, which can be attributed to co-infection of cervical tissue with other pathogens including *C. trachomatis*.

Our qPCR results show strong correlation between HHV-6 and *C. trachomatis* infection in these patients. However, currently the role of other pathogens like Herpes simplex virus 1 and 2 (HSV1/2) [47] or *Neisseria gonorrhoeae* [37], which are also regularly detected in cervical epithelial cells, remains elusive. More elaborate studies are required to understand the exact mechanism behind such co-infections and their effect on disease progression. Although HHV-6 is very frequently detected in various grades of cervical lesions, a role of HHV-6 in cervical cancer is still debatable. Because of lack of any molecular mechanism to associate HHV-6 together with other pathogens to cervical cancer, HHV-6 has been so far regarded as a bystander [48] in the oncogenesis of cervical cancer. At the same time, several studies have pointed out a strong role of HHV-6 in cervical pathogenesis [17], [31], [49], [50]. To the best of our knowledge, no in depth study has been carried out so far to look for possible mechanism and role of HHV-6 and *Chlamydia* in cervical diseases. Our study for the first time links *Chlamydia* infection to HHV-6 pathogenesis in cervical epithelial cells. More detailed study in this regard is necessary to understand the consequences of such interactions in the context of disease development.

## Supporting Information

Figure S1 Gardella gel analysis and subsequent southern hybridization showing extra-chromosomal HHV-6 DNA after *C. trachomatis* infection. HSB-ML cells were infected with *C. trachomatis* (MOI 5) for 2 days after which cells were washed thoroughly and grown in presence of fresh media with 1 µg/ml of doxycycline for another 7 days. Fresh HSB-2 cells were added to these cells and were co-cultured for another 2 weeks. 2.5×10^6^ of these cells were loaded directly onto a Gardella gel and subsequently stained with ethidium bromide and afterwards transferred to a nylon membrane. HHV-6-infected HSB-2 for productive virus infection (HSB-2+HHV-6), *C. trachomatis* elementary bodies (purified Ctr EB) and *C. trachomatis*-infected HSB-ML cells, which were subsequently not co-cultured with HSB-2 cells (HSB-ML+Ctr) were loaded as controls. Nylon membrane was hybridized with HHV-6-specific probe. Extra-chromosomal linear HHV-6 DNA and possible nicked circular viral DNA are marked.
(TIF)

Table S1 Oligos used for qPCR, southern hybridizations and cloning of plasmids for standard curve generation.
(DOCX)

Table S2 Intra- and inter-assay variability for qPCR assay. Ct, cycle threshold; SD, standard deviation; CV, coefficient of variation.
(DOCX)

Table S3 Kruskal-Wallis test to demonstrate the association between *Chlamydia* and HHV-6 load in cervical smear of all patients. Samples have been arbitrarily divided into 4 sub groups (group 1, 2, 3 and 4) depending on the HHV-6 viral load. Respective HHV-6 DNA load is mentioned within brackets. SD, standard deviation.
(DOCX)

Table S4 Kruskal-Wallis test to demonstrate the association between *Chlamydia* and HHV-6 load in cervical smear of patients with moderate to low chlamydial load. Group B: Chlamydial DNA load between 100–25000 chlamydial genome copies/1000 cells; Group C: Chlamydial DNA load below 100 genome copies (not detectable)/1000 cells. *Samples have been arbitrarily divided into 4 sub groups (group 1, 2, 3 and 4) depending on the HHV-6 viral load. Respective HHV-6 DNA load is mentioned within brackets. SD, standard deviation. Significance = 0.048.
(DOCX)

Table S5 Two-way Chi-square test to demonstrate the association between *Chlamydia* and HHV-6 load in cervical smear of patients having moderate to low chlamydial load. *Samples have been arbitrarily divided into 4 sub groups (group 1, 2, 3 and 4) depending on the HHV-6 viral load. Respective HHV-6 DNA load is mentioned within brackets. Similarly, all the samples have been arbitrarily divided into 3 sub groups (group A, B and C) depending on the chlamydial DNA load. **Group B: between 100–25000 DNA chlamydial genome copies/1000 cells; Group C: Below 100 chlamydial genome copies (not detectable)/1000 cells. Significance = 0.024.
(DOCX)

Table S6 Binomial test showing higher preference of HHV-6 activation in cervical epithelial cells than in the whole blood counterpart. Group 1, samples where HHV-6 copy number in cervical smear is more than in total blood. Group 2, samples where HHV-6 copy number in cervical smear is less than in total blood.
(DOCX)
