# Supplementary figures and images for: Chlamydia trachomatis Infection Induces Replication of Latent HHV-6
Source: PLoS One. 2013 Apr 19;8(4):e61400. doi: 10.1371/journal.pone.0061400 (PMC3631192; doi:10.1371/journal.pone.0061400)

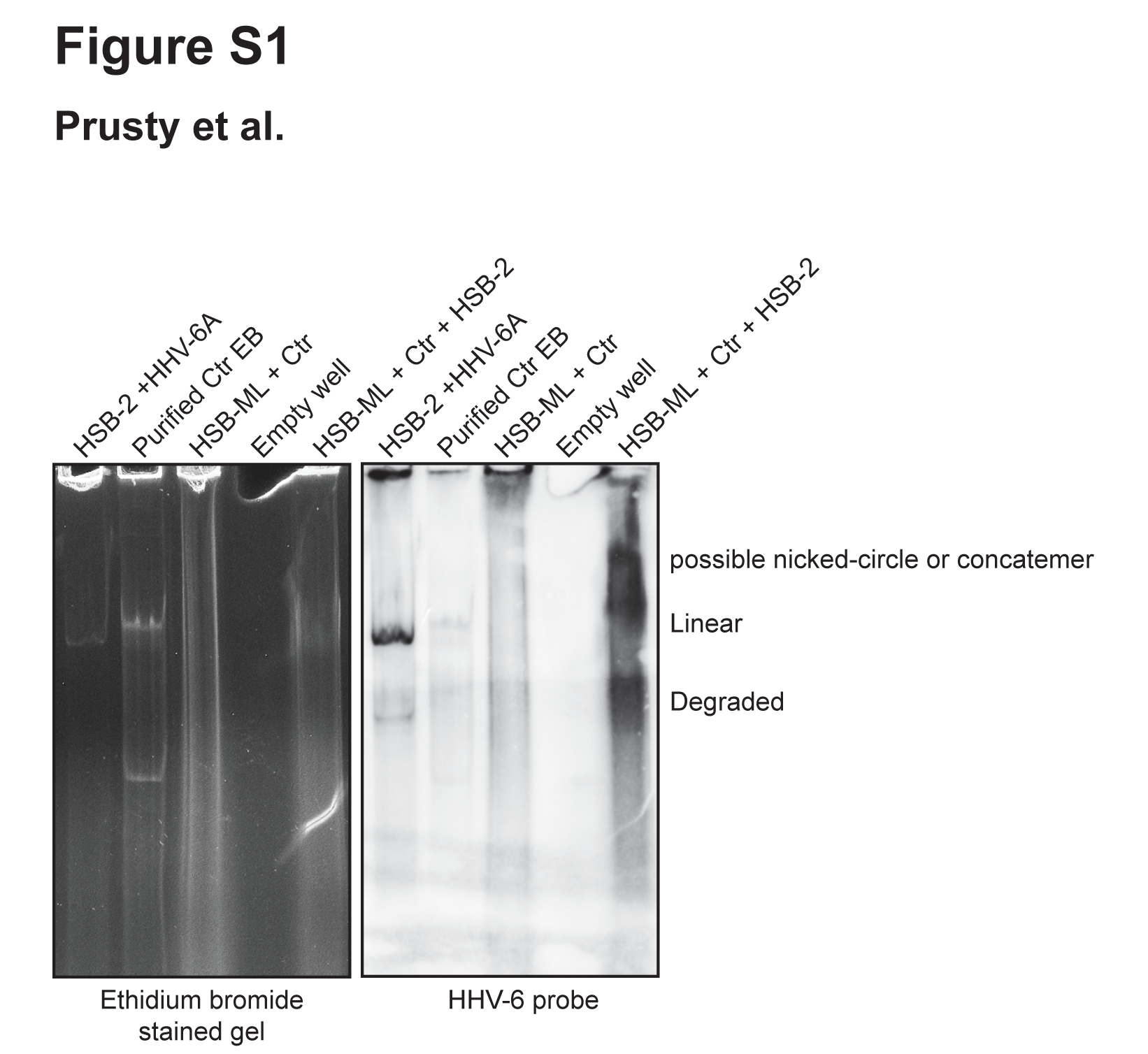

Supplement: Figure S1 — Gardella gel analysis and subsequent southern hybridization showing extra-chromosomal HHV-6 DNA after C. trachomatis infection. HSB-ML cells were infected with C. trachomatis (MOI 5) for 2 days after which cells were washed thoroughly and grown in presence of fresh media with 1 µg/ml of doxycycline for another 7 days. Fresh HSB-2 cells were added to these cells and were co-cultured for another 2 weeks. 2.5×10^6^ of these cells were loaded directly onto a Gardella gel and subsequently stained with ethidium bromide and afterwards transferred to a nylon membrane. HHV-6-infected HSB-2 for productive virus infection (HSB-2+HHV-6), C. trachomatis elementary bodies (purified Ctr EB) and C. trachomatis-infected HSB-ML cells, which were subsequently not co-cultured with HSB-2 cells (HSB-ML+Ctr) were loaded as controls. Nylon membrane was hybridized with HHV-6-specific probe. Extra-chromosomal linear HHV-6 DNA and possible nicked circular viral DNA are marked. (TIF) [file pone.0061400.s001.tif]
