# Supplementary material for: Chlamydia trachomatis Infection Induces Replication of Latent HHV-6
Source: PLoS One. 2013 Apr 19;8(4):e61400. doi: 10.1371/journal.pone.0061400 (PMC3631192; doi:10.1371/journal.pone.0061400)
Supplement: Table S1 — Oligos used for qPCR, southern hybridizations and cloning of plasmids for standard curve generation. (DOCX) [file pone.0061400.s002.docx]

| Gene | Sequence (5’ →3’) | Amplimer size |
| --- | --- | --- |
| **Primers used for preparing plasmids for Standard curve generation** | | |
| HHV-6A.U94 | ATGTTTTCCATAATAAATCCG (forward) | 1473bp |
|  | TTATAAAATTTTCGGAACCGT (reverse) |  |
| Ctr LcrH/SycD | AGGCGTACGATTTGATCGCCAA (forward) | 136bp |
|  | GCCTACATCTGCTACACCCTGC (reverse) |  |
| PI15 | ATGATAGCAATCTCTGCCGTCA (forward) | 777bp |
|  | TTTAAACCAGTACAGGTAGTTTGACGTA (reverse) |  |
| **Primers for qPCR** | | |
| HHV-6A.U94 | GCGCTCCCGGTGAGTGCATA (forward) | 110bp |
|  | AGGCCCCATGGAGTGGGAGG (reverse) |  |
| Ctr LcrH/SycD | AGGCGTACGATTTGATCGCCAA (forward) | 136bp |
|  | GCCTACATCTGCTACACCCTGC (reverse) |  |
| PI15 | GGCGGAAGCGCTACATTTCGCA (forward) | 100bp |
|  | TATTCCATATTTGCTGCCGGTGGGA (Reverse) |  |
| 5S rDNA | GTCTACGGCCATACCACCC (forward) | 121bp |
|  | AAAGCCTACAGCACCCGGT (reverse) |  |
| **Oligos for Southern Hybridization** | | |
| HHV-6 Probe | CTTACACTTGCCATGCTAGC |  |
| Ctr probe | AGGCGTACGATTTGATCGCCAA |  |
