# Supplementary material for: Chlamydia trachomatis Infection Induces Replication of Latent HHV-6
Source: PLoS One. 2013 Apr 19;8(4):e61400. doi: 10.1371/journal.pone.0061400 (PMC3631192; doi:10.1371/journal.pone.0061400)
Supplement: Table S2 — Intra- and inter-assay variability for qPCR assay. Ct, cycle threshold; SD, standard deviation; CV, coefficient of variation. (DOCX) [file pone.0061400.s003.docx]

| **HHV-6 copy number assay** | | | | | | |
| --- | --- | --- | --- | --- | --- | --- |
|  | Intra-assay variability | |  | Inter-assay variability | |  |
| Number of copies | Ct | |  | Ct | |  |
|  | Mean | SD | CV (%) | Mean | SD | CV (%) |
| 10 | 30.677 | 0.178 | 0.580239267 | 30.7325 | 0.078488853 | 0.255393647 |
| 100 | 27.6179 | 0.273 | 0.988489349 | 27.46995 | 0.209232897 | 0.761679204 |
| 1000 | 24.26 | 0.045 | 0.185490519 | 24.31 | 0.070710678 | 0.290870745 |
| 10000 | 21.077 | 0.153 | 0.725909759 | 21.099 | 0.031112698 | 0.147460535 |
| 100000 | 17.7733 | 0.069 | 0.388222784 | 17.65365 | 0.169210653 | 0.958502365 |
| **Chlamydia copy number assay** | | | | | | |
|  | Intra-assay variability | |  | Inter-assay variability | |  |
| Number of copies | Ct | |  | Ct | |  |
|  | Mean | SD | CV (%) | Mean | SD | CV (%) |
| 100 | 29.02 | 0.032 | 0.11026878 | 29.065 | 0.063 | 0.216755548 |
| 1000 | 25.952 | 0.121 | 0.46 | 26.316 | 0.114 | 0.433 |
| 10000 | 22.426 | 0.397 | 1.770266655 | 22.067 | 0.507 | 2.297548375 |
| 100000 | 19.274 | 0.312 | 1.6187 | 18.962 | 0.254 | 1.3395 |
| 1000000 | 15.988 | 0.512 | 3.202401801 | 15.511 | 0.673 | 4.338856296 |
| **Host genome number assay** | | | | | | |
|  | Intra-assay variability | |  | Inter-assay variability | |  |
| Number of copies | Ct | |  | Ct | |  |
|  | Mean | SD | CV (%) | Mean | SD | CV (%) |
| 10 | 25.968 | 0.3 | 1.155268022 | 25.899 | 0.097580736 | 0.376774145 |
| 100 | 24.631 | 0.237 | 0.962202103 | 24.4855 | 0.205768073 | 0.840367047 |
| 1000 | 21.597 | 0.059 | 0.273186091 | 21.612 | 0.021213203 | 0.098154745 |
| 10000 | 19.012 | 0.026 | 0.136755733 | 19.168 | 0.220617316 | 1.150966797 |
| 100000 | 16.078 | 0.03 | 0.186590372 | 16.2545 | 0.249608694 | 1.535628249 |
