# Supplementary material for: Chlamydia trachomatis Infection Induces Replication of Latent HHV-6
Source: PLoS One. 2013 Apr 19;8(4):e61400. doi: 10.1371/journal.pone.0061400 (PMC3631192; doi:10.1371/journal.pone.0061400)
Supplement: Table S3 — Kruskal-Wallis test to demonstrate the association between Chlamydia and HHV-6 load in cervical smear of all patients. Samples have been arbitrarily divided into 4 sub groups (group 1, 2, 3 and 4) depending on the HHV-6 viral load. Respective HHV-6 DNA load is mentioned within brackets. SD, standard deviation. (DOCX) [file pone.0061400.s004.docx]

|  | | Chlamydial load /10^3^cells |
| --- | --- | --- |
| Group 1 (<5) | Total number of samples | 38 |
|  | Mean | 2938720,0497 |
|  | Median | 6248,2902 |
|  | SD | 17782053,29463 |
| Group 2 (5-100) | Total number of samples | 18 |
|  | Mean | 25181,6152 |
|  | Median | 4287,2362 |
|  | SD | 42574,46663 |
| Group 3 (100-200) | Total number of samples | 11 |
|  | Mean | 15509,3125 |
|  | Median | 2865,9608 |
|  | SD | 20646,74488 |
| Group 4 (>200) | Total number of samples | 6 |
|  | Mean | 1225,8764 |
|  | Median | 0,0000 |
|  | SD | 2614,70362 |
| Total | Total number of samples | 73 |
|  | Mean | 1538391,6255 |
|  | Median | 2865,9608 |
|  | SD | 12831659,63386 |

*Significance = 0.236
