# Supplementary material for: Chlamydia trachomatis Infection Induces Replication of Latent HHV-6
Source: PLoS One. 2013 Apr 19;8(4):e61400. doi: 10.1371/journal.pone.0061400 (PMC3631192; doi:10.1371/journal.pone.0061400)
Supplement: Table S4 — Kruskal-Wallis test to demonstrate the association between Chlamydia and HHV-6 load in cervical smear of patients with moderate to low chlamydial load. Group B: Chlamydial DNA load between 100–25000 chlamydial genome copies/1000 cells; Group C: Chlamydial DNA load below 100 genome copies (not detectable)/1000 cells. *Samples have been arbitrarily divided into 4 sub groups (group 1, 2, 3 and 4) depending on the HHV-6 viral load. Respective HHV-6 DNA load is mentioned within brackets. SD, standard deviation. Significance = 0.048. (DOCX) [file pone.0061400.s005.docx]

|  | | | Chlamydial load /10^3^cells **(Group B and C)** |
| --- | --- | --- | --- |
| HHV-6 load* | Group 1 (<5) | Total number of samples | 23 |
|  |  | Mean | 2657,6983 |
|  |  | Median | 0 |
|  |  | SD | 5913,09569 |
|  | Group 2 (5-100) |  | 13 |
|  |  | Mean | 5301,7859 |
|  |  | Median | 2387,1410 |
|  |  | SD | 7684,52487 |
|  | Group 3 (100-200) | Total number of samples | 9 |
|  |  | Mean | 7356,1972 |
|  |  | Median | 1291,9818 |
|  |  | SD | 10062,12028 |
|  | Group 4 (>200) | Total number of samples | 6 |
|  |  | Mean | 1225,8764 |
|  |  | Median | 0 |
|  |  | SD | 2614,70362 |
|  | Total | Total number of samples | 51 |
|  |  | Mean | 3992,3786 |
|  |  | Median | 0 |
|  |  | SD | 7117,61101 |
