# Supplementary material for: Chlamydia trachomatis Infection Induces Replication of Latent HHV-6
Source: PLoS One. 2013 Apr 19;8(4):e61400. doi: 10.1371/journal.pone.0061400 (PMC3631192; doi:10.1371/journal.pone.0061400)
Supplement: Table S5 — Two-way Chi-square test to demonstrate the association between Chlamydia and HHV-6 load in cervical smear of patients having moderate to low chlamydial load. *Samples have been arbitrarily divided into 4 sub groups (group 1, 2, 3 and 4) depending on the HHV-6 viral load. Respective HHV-6 DNA load is mentioned within brackets. Similarly, all the samples have been arbitrarily divided into 3 sub groups (group A, B and C) depending on the chlamydial DNA load. **Group B: between 100–25000 DNA chlamydial genome copies/1000 cells; Group C: Below 100 chlamydial genome copies (not detectable)/1000 cells. Significance = 0.024. (DOCX) [file pone.0061400.s006.docx]

|  |  |  | **HHV-6 viral load (per 10^3^ cells)*** | | | | Total |
| --- | --- | --- | --- | --- | --- | --- | --- |
|  |  |  | Group 1  (<5) | Group 2  (5-100) | Group 3  (100-200) | Group 4  (>200) |  |
| **Chlamydial load**  **per 10^3^ cells**** | Group B | Total no. of samples | 6 | 10 | 5 | 2 | 23 |
|  |  | % of samples within the group | 26,1% | 43,5% | 21,7% | 8,7% | 100,0% |
|  | Group C | Total no. of samples | 17 | 3 | 4 | 4 | 28 |
|  |  | % of samples within the group | 60,7% | 10,7% | 14,3% | 14,3% | 100,0% |
| Total | | Total no. of samples | 23 | 13 | 9 | 6 | 51 |
|  | | % of samples within the group | 45,1% | 25,5% | 17,6% | 11,8% | 100,0% |
