# Supplementary material for: Chlamydia trachomatis Infection Induces Replication of Latent HHV-6
Source: PLoS One. 2013 Apr 19;8(4):e61400. doi: 10.1371/journal.pone.0061400 (PMC3631192; doi:10.1371/journal.pone.0061400)
Supplement: Table S6 — Binomial test showing higher preference of HHV-6 activation in cervical epithelial cells than in the whole blood counterpart. Group 1, samples where HHV-6 copy number in cervical smear is more than in total blood. Group 2, samples where HHV-6 copy number in cervical smear is less than in total blood. (DOCX) [file pone.0061400.s007.docx]

|  | | Number of samples | % of total samples | Test share | Exact significance (2-sided) |
| --- | --- | --- | --- | --- | --- |
|  | Group 1 | 34 | 0.97 | 0.50 | 0.000 |
| Proportion | Group 2 | 1 | 0.03 |  |  |
|  | Total | 35 | 1.00 |  |  |
